# *Echinococcus multilocularis* (Cestoda, Cyclophyllidea, Taeniidae): oncospheral hook morphogenesis

**DOI:** 10.1007/s00436-016-5131-1

**Published:** 2016-05-20

**Authors:** Zdzisław Świderski, Jordi Miquel, Samira Azzouz-Maache, Anne-Françoise Pétavy

**Affiliations:** 1 W. Stefański Institute of Parasitology, Polish Academy of Sciences, 51/55 Twarda Street, 00-818 Warszawa, Poland; 2 Secció de Parasitologia, Departament de Biologia, Sanitat i Medi Ambient, Facultat de Farmàcia i Ciències de l’Alimentació, Universitat de Barcelona, Av. Joan XXIII, sn, 08028 Barcelona, Spain; 3 Institut de Recerca de la Biodiversitat (IRBio), Facultat de Biologia, Universitat de Barcelona, Av. Diagonal, 645, 08028 Barcelona, Spain; 4 Laboratoire de Parasitologie et Mycologie Médicale, Faculté de Pharmacie, Université Claude Bernard-Lyon 1, 8 Av. Rockefeller, 69373 Lyon Cedex 08, France

**Keywords:** *Echinococcus multilocularis*, Taeniid cestodes, Preoncospheral differentiation, Oncospheral hook morphogenesis, Oncoblasts, Hook-forming centre, Ultrastructure

## Abstract

Ultrastructural characteristics of the oncospheral hook morphogenesis in the taeniid cestode *Echinococcus multilocularis* Leuckart, 1863, a parasite of medical and veterinary importance, are described. Oncospheral hook primordia appear at the preoncospheral phase of the embryonic development. Within six specialised cells of the so-called oncoblasts, high concentration of mitochondria, numerous ribosomes and extended Golgi regions are involved in hook development. During hook growth, the blade and base gradually protrude outside the oncoblast plasma membrane. The nucleated oncoblast persists around the handles of fully formed hooks. Simultaneously with the hook primordium elongation and transformation into a blade, handle and base, the hook material differentiates into an electron-dense cortex and a less dense inner core. The exit of the blade of each mature hook, protruding from the oncosphere, is surrounded by a circular, septate desmosome and two rigid, dense rings on either side. The pattern of oncospheral hook morphogenesis in *E. multilocularis* is compared with that of other previously examined cyclophyllidean cestodes. Though oncoblasts have never been observed around the mature hooks, their remnants are often still visible in the fully developed infective oncospheres in particular in some taeniid species so far examined in this respect. The origin and formation of oncospheral hooks in *E. multilocularis*, evidently differs from that of the rostellar hooks. Thus, although the hooks may have slight similarity at the gross level, they are neither analogous nor homologous structures.

## Introduction

The oncospheral hooks of *Echinococcus multilocularis* hexacanths, together with penetration gland secretion, play an important and active role during hexacanth penetration of the intestinal tissue of its intermediate hosts, humans and wild rodents. Despite numerous light and electron microscopical studies on different cestode species (for references, see Ogren 1955, 1957, 1958, 1961; Rybicka 1966; Świderski 1967, 1973, 1983; Ubelaker, 1983; Świderski and Tkach, 1997a, b; Świderski et al. 2000a, b, 2004; Młocicki et al. 2005), nothing is known about the ultrastructural details of oncospheral hook morphogenesis among cestodes of the family Taeniidae. The ultrastructure of mature oncospheral hooks was briefly described only in two taeniid species: *Taenia taeniaeformis* by Nieland (1968) and *Taenia crassiceps* by Chew (1983).

The purpose of this paper is to describe ultrastructural aspects of oncospheral hook morphogenesis in the taeniid cestode *E. multilocularis*, a parasite of medical and veterinary importance.

## Materials and methods

### Materials

Live specimens of *E. multilocularis* were isolated from the intestine of a naturally infected red fox (*Vulpes vulpes* L.) from La Roche sur Foron (France) captured in June 2014.

### TEM preparation of samples

Adult-recovered tapeworms were immediately rinsed with a 0.9 % NaCl solution. Later, they were fixed in cold (4 °C) 2.5 % glutaraldehyde in a 0.1 M sodium cacodylate buffer at pH 7.4 for a minimum of 2 h, rinsed in 0.1 M sodium cacodylate buffer at pH 7.4, post-fixed in cold (4 °C) 1 % osmium tetroxide with 0.9 % potassium ferricyanide in the same buffer for 1 h, rinsed in Milli-Q water (Millipore Gradient A10), dehydrated in an ethanol series and propylene oxide, embedded in Spurr’s resin and polymerised at 60 °C for 72 h.

Ultrathin sections (60–90-nm thick) of mature segments at the level of the vas deferens were obtained in a Reichert-Jung Ultracut E ultramicrotome. Sections were placed on 200-μm mesh copper grids and double-stained with uranyl acetate and lead citrate according to the Reynolds (1963) methodology. The grids were examined in a JEOL 1010 transmission electron microscope (Jeol, Japan) operated at 80 kV, in the “Centres Científics i Tecnològics” of the University of Barcelona (CCiTUB).

### Freeze substitution and infiltration with Lowicryl resin

Some specimens were fixed in cold (4 °C) 4 % paraformaldehyde + 0.1 % glutaraldehyde in a 0.1 M sodium cacodylate buffer at pH 7.4 for a 4 to 5 h and then conserved in cold (4 °C) 2 % paraformaldehyde in the same buffer. Samples were rinsed in a 0.15 M glycine in a 0.1 M sodium cacodylate buffer at pH 7.4, cryoprotected by crescent concentrations (10, 20 and 30 %) of glycerol in the same buffer, and then cryofixed in liquid propane.

Samples were freeze-substituted for 3 days at −90 °C in anhydrous acetone containing 0.5 % uranyl acetate. Then, they were warmed up to −50 °C, at 5 °C/h (EM AFS2, Leica, Vienna, Austria). After several acetone rinses, samples were infiltrated with Lowicryl HM20 resin during 4 days. Samples were polymerised under UV light at −50 °C for 24 h, during the warming up at a rate 5 °C/h until 22 °C and 48 h at 22 °C.

Ultrathin sections were picked up on Formvar-coated niquel grids, double-stained with uranyl acetate and lead citrate and examined in a JEOL 1010 TEM operated at 80 kV, in the CCiTUB.

### Cytochemistry

The periodic acid-thiosemicarbazide-silver proteinate (PA-TSC-SP) technique of Thiéry (1967) was applied to determine the cytochemical localisation of glycogen at the ultrastructural level. Thus, ultrathin sections collected on gold grids were treated as follows: 30 min in 10 % PA, rinsed in Milli-Q water; 24 h in TCH, rinsed in acetic solutions; and Milli-Q water, 30 min in 1 % SP in the dark and rinsed in Milli-Q water. Gold grids were also examined in a JEOL 1010 TEM operated at an accelerating voltage of 80 kV, in the CCiTUB.

## Results

**Fig. 1:**
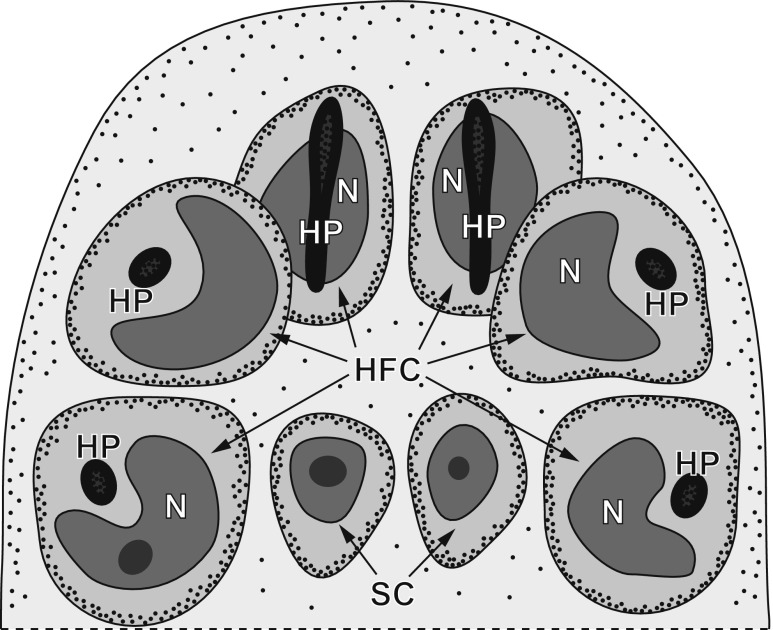
Diagram of the general topography of the anterior pole of the *Echinococcus multilocularis* embryo in the stage of hook formation (preoncospheral phase of embryogenesis). Note six hook-forming cells or oncoblasts with differentiating hook primordia and bilaterally symmetrical pattern of blastomere arrangement. *HFC* hook-forming cells, *HP* hook primordia, *N* nucleus, *SC* somatic cells

**Fig. 2:**
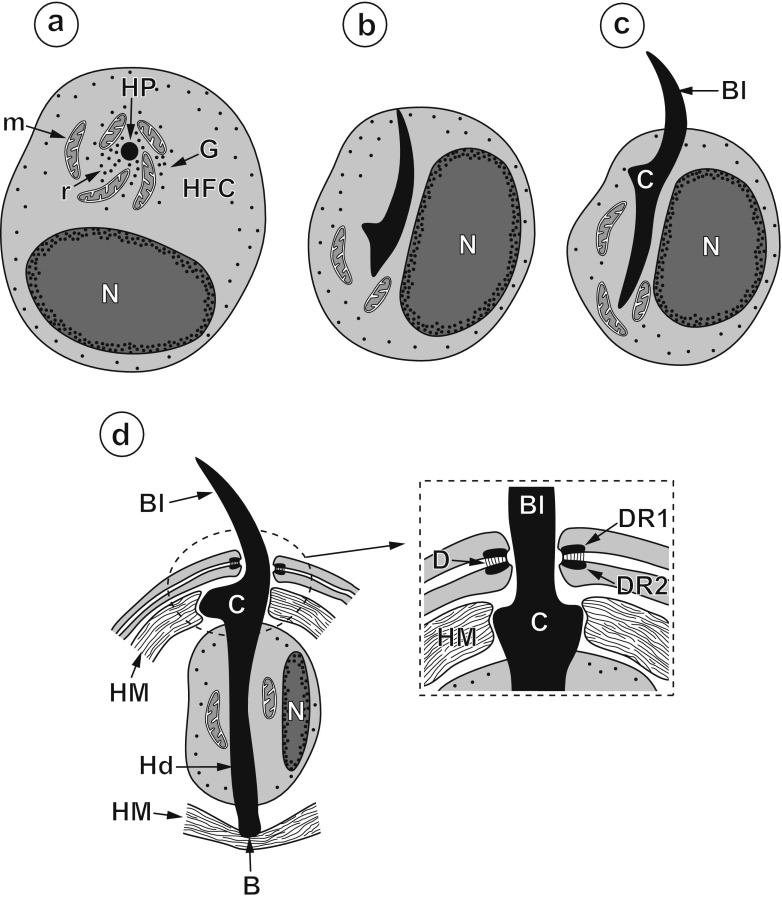
Diagram of four consecutive stages of oncospheral hook development. **a** Early oncoblast with hook primordium surrounded by an accumulation of cell organelles of the hook-forming centre. **b** Early oncoblast with intracellular outline of blade still embedded in its cytoplasm. **c** Late oncoblast with the blade protruding outside and the early handle formation. **d** Mature oncospheral hook with degenerating oncoblast surrounding the handle. *Inset*: The enlarged detail of hook exit surrounded by a circular septate desmosome and two electron-dense rings at its both sides. *B* hook base, *Bl* hook blade, *C* hook collar, *D* circular septate desmosome, *DR1* and *DR2* dense rings situated at both sides of the circular septate desmosome, *G* Golgi regions, *Hd* hook handle, *HFC* hook-forming cells, *HM* hook muscles, *HP* hook primordium or hook anlage, *m* mitochondria, *N* nucleus, *r* ribosomes and polyribosomes

**Fig. 3:**
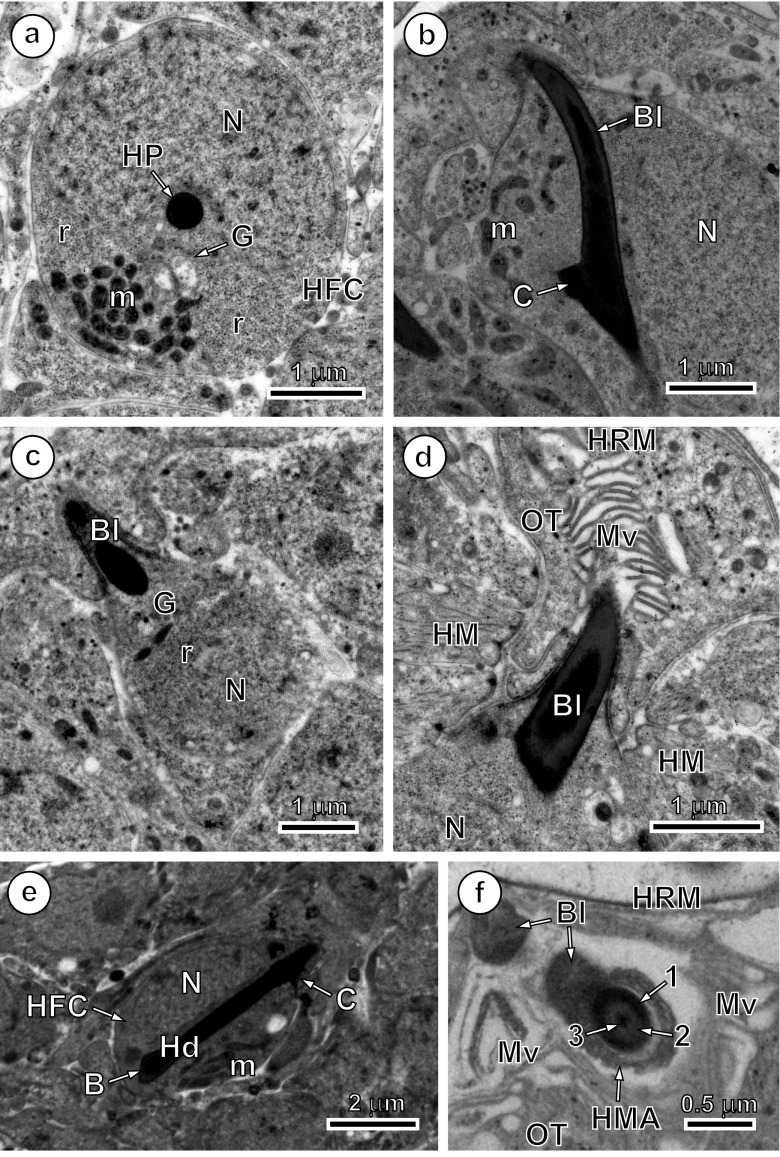
Transmission electron micrographs of the consecutive stages of oncospheral hook morphogenesis. **a** Early oncoblast with kidney-shaped nucleus and hook primordium situated in its invagination. Note the so-called hook-forming centre surrounding initially spherical, electron-dense hook primordium and composed of a dense accumulation of numerous mitochondria, ribosomes and/or polyribosomes and Golgi regions. **b** Early stage of blade elongation still surrounded by oncoblast plasma membrane and before its protruding from hook-forming cell. Note that the sharp ending of the hook blade touches the cell membrane in only one point and never follows its shape while both the blade and elongating hook handle or shank is closely adjacent to oncoblast nucleus remaining in its kidney-shaped invagination. **c** Longitudinally oblique section showing advanced stage of blade elongation just before its protruding from the oncoblast. **d** Oblique section through the region of blade exit into a hook region peripheral cavity situated between the oncospheral tegument with numerous long microvilli and the so-called hook region membrane. **e** Hook handle or shank entirely surrounded by the nucleated hook-forming cell showing several mitochondria in its cytoplasm; the hook collar and hook base are visible on both extremities if hook handle. **f** Oblique section through the curved part of hook blade in the hook region peripheral cavity situated between the oncospheral tegument with tegumental microvilli and the hook region membrane. Observe a moderately electron-dense, crescent-shaped zone of hook muscle attachment around the lower part of the hook blade enlarged into a hook collar. Note that in the hook material, 3 or 4 layers of different electron density can be noticed (on Fig. 3f only 1–3) in the different part of hooks, but its heterogeneity divided into an outer cortex and an inner core is continued along the entire length of the hook, corresponding to the outer core, middle layer and inner cortex, can be distinguish on the section. *B* hook base, *Bl* hook blade, *C* hook collar, *G* Golgi regions, *Hd* hook handle, *HFC* hook-forming cell, *HMA* hook muscle attachment, *HP* hook primordium, *HRM* hook region membrane, *m* mitochondria, *Mv* microvilli, *N* nucleus, *OT* oncospheral tegument, *r* ribosomes and polyribosomes

In *E. multilocularis*, six oncospheral hooks are formed in six specialised cells of the so-called oncoblasts (Ogren 1955) during the early preoncospheral phase of embryonic development. The symmetrically arranged hook-forming cells are localised at one pole of the differentiating embryo which shows already a bilateral symmetry of differentiating blastomeres (Fig. 1). The consecutive stages of oncospheral hook morphogenesis are illustrated in Figs. 2 and 3. The oncoblasts are characterised by an early displacement of their large, kidney-shaped nuclei into one pole of the cell; it represents the first sign of their differentiation. At the opposite pole of each oncoblast appears simultaneously, the so-called hook-forming centre composed of a high concentration of mitochondria, numerous ribosomes and extended Golgi regions, all of which are apparently involved in hook development. The hook primordium appears initially within the oncoblast cytoplasm as a large electron-dense granule (Figs. 2a and 3a), which subsequently undergoes progressive elongation (Figs. 2b and 3b). The hooklet which is transformed into the blade (Figs. 2b and 3b, c) serves as the focal point for further growth and differentiation of the hook; the elongating blade is always closely adjacent to the concave surface of the nuclear membrane and never follows the oval shape of the oncoblast plasma membrane (Figs. 2b and 3b, c).

With handle elongation, the blade projects beyond the cellular membrane of the oncoblast and becomes surrounded by a thick cytoplasmic sheath (Fig. 2c, d). Simultaneously, the anterior part of the hook undergoes further differentiation (Figs. 2 inset and 3c, d). The two membranes situated between the cytoplasmic sheath and the oncoblast surface take part in the formation of a circular septate desmosome (Figs. 2 inset, 3d and 4), which surrounds the hook blade at the site of its exit from the oncosphere.

**Fig. 4:**
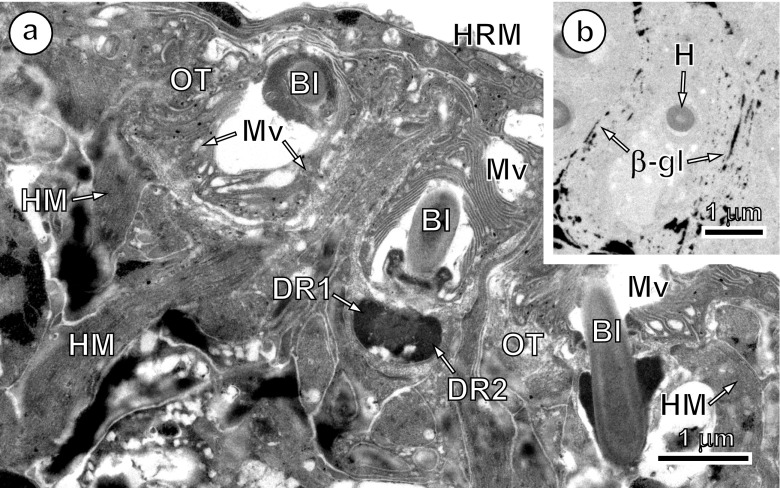
Details of oncospheral hook exit, hook region membrane and cytochemistry of glycogen in hook muscles. **a** A section through the hook region of the oncosphere showing three blade exits into a peripheral cavity situated between the oncospheral tegument with numerous long microvilli and the so-called hook region membrane. Note the characteristic dense rings, always localised on both sides of circular, septate desmosomes, which always accompany oncospheral hook exits and numerous bundles of hook muscles associated with hook collars. **b** Cytochemical test of Thiéry showing positive reaction for numerous particles of β-glycogen in the peripheral layers of hook muscles. *β*-*gl* β-glycogen, *Bl* hook blade, *DR1* and *DR2* dense rings, *HM* hook muscles, *HRM* hook region membrane, *Mv* microvilli, *OT* oncospheral tegument

When the hook formation is completed, the so-called hook region membrane (Świderski 1972) is formed where the hook blades protrude from the oncosphere surface (Figs. 3d, f and 4). This structure is well defined in *E. multilocularis* and consists of a membrane-bound cytoplasmic layer that surrounds the hook blades in a cap-like manner only at one pole of the oncosphere. Microvilli are especially well-developed on the surface of the oncospheral tegument covered by the hook region membrane (Figs. 3d, f and 4). Cytochemical test of Thiéry (1967) at ultrastructural level always gave strongly positive reaction for β-glycogen particles in the peripheral layers of the hook muscles (Fig. 4b).

Mature oncospheral hooks of *E. multilocularis* are differentiated into two pairs of lateral and a pair of median hooks. The lateral hooks are more robust than the median ones. The fully formed hooks in cross-, longitudinal and oblique sections (Figs. 3f and 4) appear heterogeneous bipartite structures composed of a high electron-dense outer sheath or cortex and a less electron-dense inner core. The collar region shows tripartite structure on thin sections (Figs. 3f and 4) due to slightly electron-dense material forming a large ring around a high electron-dense outer sheath of the enlarged guard. This heterogeneity of hook material, of outer cortex and an inner core is continued along the entire length of the hook.

## Discussion

In *E. multilocularis*, as in other cestodes, the oncospheral hooks and penetration gland of the larva play an important role and apparently cooperate during the hexacanth invasion of the intermediate host. The importance of both structures in the infection mechanism has been reported in numerous studies (for review, see Rybicka 1966; Świderski 1973, 1976; Conn 1991; Ubelaker 1983; Świderski et al. 2000a, b).

Most details of hook formation are beyond the resolution of light microscopy; this may explain much misleading information in early papers based only on optical methods. However, the first light microscopical studies of the preoncospheral phase of embryonic development (Ogren 1955, 1957, 1958, 1961; Świderski 1967, Moczoń 1971) indicated clearly that hook morphogenesis takes place intracellularly, inside specialised blastomeres, of the so-called oncoblasts (Ogren 1955). In all cestode species examined in this respect, the oncoblasts occur in grouped of three pairs: one medial and two lateral, and are always symmetrically arranged near the anterior pole of the preoncosphere. All consecutive stages of hook formation inside the oncoblasts were first described by Ogren (1961) on *Hymenolepis diminuta* and confirmed more recently by TEM studies (Świderski 1973, 1976, 1983; Świderski and Tkach 1997a; Świderski et al. 2000a, b). Ogren (1961) distinguished five stages of hook development: (1) early oncoblast, initiating hook synthesis; (2) early oncoblast, with blade outline completed; (3) late oncoblast during shank synthesis; (4) late oncoblast with shank completed and (5) oncoblast degeneration when the fully developed hook is completely formed. In *E. multilocularis*, similar stages were observed, but the formation of the hook base and the process of hook muscle attachment were not examined in detail. Nevertheless, some details described in the early light microscopical studies (Ogren 1957, 1958, 1961; Moczoń 1971) appear incorrect, due to low resolution, and are not supported by more recent TEM studies in a few other tapeworm species by Świderski (1973, 1976, 1983), Świderski and Tkach (1997a), Kornakova (1999) and Świderski et al. (2000a, b).

The first electron microscopical and histochemical analysis of oncospheral hook morphogenesis were in *Catenotaenia pusilla* and concerned factors determining hook shape as well as chemical aspects of hook formation (Świderski 1973). These results suggested that the shapes of hooks are determined genetically and not governed by the profile of the oncoblast plasma membrane, as suggested by Ogren (1961). Also in *E. multilocularis*, the oncoblast membrane does not seem to determine or mould hook shape, since it does not closely follow the hook shape and only touched the hook blade at one point.

The present study indicates that hook musculature of *E. multilocularis*, containing numerous β-glycogen particles, is directly attached only to the hook collar and base, not to the surface of the oncoblast. Similar attachment of hook muscle was also observed in *Inermicapsifer madagascariensis* by Świderski (1976), *Pseudhymenolepis redonica* by Tkach and Świderski (1997), *Nematotaenia dispar* by Świderski and Tkach (1997a), *Dilepis undula* by Świderski et al. (2000a), *Hepatocestus hepaticus* by Świderski et al. (2000b), *Anoplocephaloides dentata* by Świderski et al. (2001) and *Joyeuxiella echinorhyncoides* by Świderski et al. (2004).

Our studies differ in significant ways from that of Kornakova (1999) on the morphogenesis of oncospheral hooks in *Passerilepis crenata*, a hymenolepidid from passerine birds. She indicated that the fully developed oncospheral hooks do not protrude through the oncoblast plasma membrane so that the entire hook-forming cell always remains intact. As discussed in detail by Świderski et al. (2000a; which see), such an arrangement is contrary to what had been observed in all similar studies at the time and remains true in the present study. Such an arrangement of hooks would seem to render an oncosphere non-functional. It is difficult to explain how the hook muscle system would function if the entire hook remains embedded in the oncoblast, including both its hook muscle attachment points at the hook collar and base. Clearly, the hooks must emerge from the formative cells at some time to become functional. The exact mechanism for this process has not been demonstrated conclusively, but may involve a specialised form of exocytosis that would result in externalisation of the hook while leaving the oncoblast intact. In *E. multilocularis*, as in other species examined to date (Collin 1968; Świderski 1973, 1976; Chew 1983; Świderski and Tkach 1997a; Tkach and Świderski 1997; Świderski et al. 2000a, b), the hook muscle system forms a very complex pattern of muscle arrangements and attachment zones. In all species examined, the muscles are attached only to the guards or collars and bases of hooks.

Our data demonstrate that the oncoblasts are characterised by high synthetic activity, based on the accumulation of free ribosomes, polyribosomes, Golgi complexes and mitochondria. Energy supplied by mitochondria is necessary for protein synthesis, which supplies construction materials for the process of hook formation (Nieland 1968; Świderski 1973, 1976). Kornakova (1999) has apparently overlooked the synthetic role of mitochondrial energy metabolism in hook formation for she does not believe the hypothesis of a possible active role of mitochondrial accumulation in hook-forming cells.

Despite some similarities in the general pattern of hook morphogenesis in cestodes, differences are evident among different species. Though oncoblasts have never been observed around the mature, fully developed hooks of *I. madagascariensis*, *Echinococcus granulosus*, *N. dispar*, *H. hepaticus* and *Mosgovoyia ctenoides* (Świderski 1976, 1983; Świderski and Tkach 1997a; Świderski et al. 2000b; Młocicki et al. 2005, respectively). Their remnants, however, are often still visible in the fully developed infective oncospheres of some species as observed in the present study on *E. multilocularis*. Other examples of the nucleated oncoblasts surrounding the hooks in infective oncospheres were described in the dilepidid tapeworm *D. undula* and in the dipylidiid cestode, *J. echinorhyncoides* by Świderski et al. (2000a, 2004). Similar observations were also reported previously by Collin (1968), Moczoń (1971), Furukawa et al. (1977), Chew (1983) and Tkach and Świderski (1997). In the fully developed oncospheres of *Staphylocystoides stefanskii*, only the thin layers of anucleated cytoplasm around the hook shank regions remain from the oncoblast. That condition seems to be a common feature for mammalian hymenolepidids (Świderski and Tkach 1997b, 1999). However, in this kind of interspecific comparison, two criteria must be taken under consideration: (1) the degree of oncosphere and hook development and (2) the presence of representative cross, oblique and longitudinal sections along the hook shank, blade and base. The cases of total degeneration of oncoblasts, as observed in *C. pusilla*, showing only six nuclei in fully developed oncospheres (Świderski 1972, 1973), are characteristic features associated with evolutionary trends in tapeworm larval simplification. Świderski (1983) has formulated the hypothesis that progressive reduction in the number of oncospheral cells, as in *C. pusilla*, is an adaptative feature in cestode evolution.

The origin and formation of oncospheral hooks in *E. multilocularis*, evidently differs from that of the rostellar hooks (Mount 1970), as may be concluded from the results of the present study and those of Świderski (1973, 1976), Świderski et al. (2000a) and Młocicki et al. (2005). The rostellar hooks of *T. crassiceps* as described by Mount (1970) originate from a fusion of specialised tegumental microtriches and a progressive deposition of proteins on the differentiating rostellar hook surface. Rostellar hooks are not individually connected to myofibrils and function in attachment to the host. Conversely, oncospheral hooks are directly attached to myofibrils and function in host invasion. Thus, although the hooks may have slight similarity at the gross level, they are neither analogous nor homologous structures.
